# The Grand Rendezvous

**Published:** 1874-01

**Authors:** 


### THE GRAND RENDEZVOUS.

An old man was dying the other day, aged seventy-two years, conscious to the very last moment, and though too weak to speak, he took the hand of his wife, and sister, and son, and daughter, successively, and mutely pointed upward, as if to say, with his latest breath, "Meet me in heaven." What a blessed thing it must be to be a Christian—to rest all hopes in the Bible promises.

When the infidel Hume was dying he called for a pack of cards, to while away the moments in playing whist. When Walter Scott was near his end, he said to Lockhart, "Bring the book."

"What book?"

"There is but ONE book," as he pointed to the family Bible that laid on the stand, as if he thought that what was in that book was the only thing in the world that was of any worth in a dying hour. Within a month we were in the room where the great novelist breathed his last, with its outlook on pastures green and fields of waving grain, and forest trees of luxuriant growth, with the clear waters of the winding Tweed, as they flowed onward to the shoreless sea, and we wondered if in his last days he did not often cast his eyes abroad and think, and bring to his memory the beautiful lines which so few Scotchmen have failed to sing many a time and oft:

"On Jordan's stormy banks I stand,
And cast a wistful eye
On Canaan's fair and happy land,
Where my possessions lie.
There everlasting spring abides,
And never withering flowers;
Death like a narrow sea divides
This heavenly land from ours."

Towards the very last hours of life, when death is gradually approached, there is ever a dreadful feeling of utter helplessness and sinking away, and there is a universal craving for some support—for something to lean upon. The last words of the great commoner, Henry Clay, were, "Don't leave me, my son." Prince Albert, of England, was one of the most accomplished gentlemen in Europe—cultivated, refined, illustrious. On his dying bed he said, "I have had wealth, rank and power; but if this were all I had how wretched should I be now," and then exclaimed:

"Rock of ages, cleft for me,
Let me hide myself in thee."

Now, reader! on which of the three are you making your calculations to lean when the last hour comes to you? on the truth of the Bible? on the hope of reunion in heaven with the dear ones left behind you? or on a pack of cards? The hope of better things to come is a better anodyne for pain than any opiate ever given; it is a balm for every wound, a cordial for every fear; but to die hopeless of heaven, with nothing to lean upon, how inconceivably dreadful!

